# A Cross-Sectional Study of Tuberculosis Knowledge, Attitude, and Practice Among the General Population in the Western Region of Saudi Arabia

**DOI:** 10.7759/cureus.29987

**Published:** 2022-10-06

**Authors:** Mohammed E Almalki, Fahad S Almuqati, Riyad Alasmari, Mohmmed J Enani, Ammar A Bahwirith, Anas A Alloqmani, Abdulmohsen Alqurashi, Abdurahman Hassan-Hussein

**Affiliations:** 1 Medicine, Umm Al-Qura University, Mecca, SAU; 2 Community Medicine, Umm Al-Qura University, Mecca, SAU

**Keywords:** respiratory disease, hajj, public health, infection control, tuberculosis

## Abstract

Background

Religious gatherings like the Hajj, an Islamic pilgrimage, attract millions of people to one place during the same time frame. Due to crowding, infectious diseases, specifically tuberculosis (TB), are very common during such events. This study investigates the knowledge, attitudes, and practices of the public in the western region of Saudi Arabia related to TB to better understand the situation.

Methodology

An observational, questionnaire-based, cross-sectional study was conducted over two months between January and March 2022. A survey of 29 questions was used to collect data from the general population. The study included any person who was a resident of Makkah. Individuals under 18 years of age and health workers were excluded. We used OpenEpi, version 3.0, for sample size calculation, which gave a result of 604 participants, and SPSS version 25.0 (IBM Corp., Armonk, NY, USA) was used for data analysis.

Results

A total of 604 participants were included in this study; 64.7% of respondents showed poor overall knowledge, and 14.1% had good knowledge of TB. Concerning attitude, 89.9% of the respondents showed poor attitude, and only 2.3% had a good attitude. As for practice, 59.4% of respondents had poor knowledge of proper practices, and only 10.4% knew the right practices regarding TB. Upon further analysis of our results, women exhibited better knowledge of TB than men (0.62, 95% confidence interval (CI), 0.44-0.87). Participants over 50 years old had the lowest knowledge about TB compared with participants aged 18 to 28 years old (7.61, 95% CI, 4.35-13.32). Non-Saudi residents had less knowledge compared with Saudi residents (45.849, 95% CI, 18.475-113.78). Level of education also played a substantial role; university graduates had the most knowledge about TB compared with participants with below university or no formal education (0.052, 95% CI, 0.01-0.40).

Conclusions

Participants with lower educational backgrounds were the most lacking in knowledge, attitude, and practice regarding TB. This lack of knowledge was more common among non-Saudi men over 50 years old. Information campaigns are needed to help reduce the prevalence of TB.

## Introduction

Tuberculosis (TB) is a well-known contagious infection that spreads through the inhalation of droplets produced by the coughs or sneezes of an infected person. TB usually affects the lungs (pulmonary TB) but can also affect multiple other organs. In 2020, the number of people infected with TB was estimated to be 9.9 million worldwide, with a drop of 18% from 2019 and an increase in mortality rate. Mortality reached almost 1.3 million deaths among human immunodeficiency virus (HIV)-negative patients from 1.2 million in 2019 [1]. In Saudi Arabia, 64,345 cases were reported over a period of 20 years; persons of non-Saudi nationalities represented 48%, and Makkah had the highest incidence rate [2]. This high incidence rate is due to the Hajj (Islamic pilgrimage) season when more than two million Muslim pilgrims visit Makkah from around 180 countries and stay for five to six days [3]. Furthermore, many visitors come from TB-endemic areas to worship and practice their rituals under circumstances believed to increase the risk of TB transmission [4]. Consequently, TB infection is incredibly high during the Hajj season; it is the most common cause of pneumonia leading to hospitalization [5]. Knowledge gaps have been detected worldwide regarding the long-term symptoms of TB and some essential preventive measures. Studies have shown that awareness of the risk of TB transmission associated with mass gatherings similar to Hajj was generally inadequate [6]. In addition, a study from Saudi Arabia among healthcare workers during Hajj found some knowledge gaps and even poor TB management practices [7].

The high incidence of TB cases coupled with the lack of knowledge globally and locally highlight the importance of population awareness, especially in regions such as Makkah, and bring forward the need for research to investigate the level of awareness among the general population. Locally, not enough studies have evaluated the knowledge of TB. In this paper, we provide an investigation of knowledge, attitudes, and practices of TB among the public in the western region of Saudi Arabia.

## Materials and methods

This observational, cross-sectional study was conducted using a self-administered questionnaire. The study targeted the general population of the western region of Saudi Arabia and spanned two months between January and March 2022. We included all Makkah residents. Individuals younger than 18 years of age and health workers were excluded from the study. Participants were selected using a non-sampling convenience sampling technique. Based on a review of similar studies, the research team developed a 29-item questionnaire divided into four sections to accomplish the research objectives [8]. The first section (six questions) focused on sociodemographic characteristics (sex, age group, nationality, educational level, monthly income, chronic diseases). The second section (10 questions) covered TB knowledge and awareness. The third section (eight questions) assessed attitudes and healthcare-seeking behaviors related to TB. The fourth section (five questions) assessed TB stigma in the community. The data were collected through an online Google survey distributed through social media platforms. Participants completed the survey anonymously and voluntarily. The questionnaire was designed in English and translated into Arabic to fit the country’s native language. We used OpenEpi (version 3.0) for sample size calculation: a minimum sample size of 385 was required for the study, considering a 95% confidence interval (CI) and anticipated frequency of 50%, and design effects of 1.

Data analysis

After data extraction and coding, SPSS Statistics version 25.0 (IBM Corp., Armonk, NY, USA) was used for data analysis. Descriptive analysis was utilized and expressed as frequency and percentage for categorical variables. Participants’ overall knowledge, attitude, and practice were categorized according to Bloom’s cutoff point into good, moderate, and poor based on the total percentage score as follows: good for scores 80% and above, moderate for scores 60-79%, and poor for scores below 60% [9]. We used univariate logistic regression to explore variables that predict poor TB knowledge. Variables identified with a P-value less than 0.25 in univariate analysis were included in the multivariate logistic regression analysis. A stepwise multiple logistic regression was used with variable entry and removal at P-values less than 0.25 and equal to or over 0.10, respectively.

Ethical considerations

This study was approved by the Medical Research Ethics Committee at Umm Al-Qura University (approval number: HAPO-02-k-012-2021-12-886). Consent to participate was taken from participants electronically.

## Results

The survey included 604 participants. The sociodemographic characteristics are presented in Table 1. Of the participants, females were predominant (52%). Most participants were in the age groups of 40-50 and 18-28 years (34.3% and 33.6%, respectively), and Saudi nationals accounted for 65.2% of the participants. Additionally, 3.1% of the participants had no formal education, 4.5% had received education at the level of elementary school, 9.4% had intermediate school education, 26.2% had high school education, and 56.8% were university graduates (Table 1).

**Table 1:** Sociodemographic characteristics of study participants.

Characteristics	Number	Percentage
Sex		
Male	290	48.0
Female	314	52.0
Age (years)		
18–28	203	33.6
29–39	81	13.4
40–50	207	34.3
Over 50	113	18.7
Nationality		
Saudi	394	65.2
Non-Saudi	210	34.8
Educational level		
No formal education	19	3.1
Elementary school	27	4.5
Intermediate school	57	9.4
High school	158	26.2
University graduate	343	56.8
Monthly income (Saudi Riyals)		
Less than 1,000	138	22.8
1,000–3,000	79	13.1
3,001–7,000	101	16.7
7,001–10,000	156	25.8
More than 10,000	130	21.5
Presence of chronic diseases		
Yes	166	27.5
No	438	72.5

Results regarding participants’ knowledge showed that only 65.6% had heard about TB, with the majority relying on family and friends and media as their primary source of information (35.4% and 34.8%, respectively). We found that 64.7% of the participants had overall poor knowledge, 21.2% had moderate knowledge, and 14.1% had good knowledge of TB. Additional data on participant knowledge are presented in Table 2.

**Table 2:** Participants’ knowledge of tuberculosis. TB: tuberculosis

Knowledge	Number	Percentage
Have heard of TB		
Yes	396	65.6
No	208	34.4
Source of information		
Newspapers and magazines	69	17.4
Media	138	34.8
Brochures, posters, etc.	107	27
Health workers	126	31.8
Family, friends	140	35.4
Teachers	83	21
Others (movies, prior exposure, academia)	24	6.1
Cause of TB		
Bacteria/germs	290	48
Cold air	89	14.7
Shortage of food	147	24.3
Smoking	93	15.4
Dust	112	18.5
Sunlight	55	9.1
Hot climate	73	12.1
Signs/symptoms of TB		
Cough for two weeks	272	45
Sputum with blood	251	41.6
Weight loss	234	38.7
Loss of appetite	217	35.9
Fever and sweating	205	33.9
Chest pain	208	34.4
Don’t know	44	7.3
Can TB be transmitted?		
Yes	310	51.3
No	175	29.0
Don’t know	119	19.7
TB transmitted through		
Handshaking	135	22.4
Cough/breath	260	43
Sharing cups	197	32.6
Sharing utensils	208	34.4
Touching items in public	173	28.6
Don’t know	58	9.6
Can TB be prevented?		
Yes	326	54.0
No	155	25.7
Don’t know	123	20.4
Preventive methods		
Avoid shaking hands	151	25
Cover mouth	259	42.9
Avoiding sharing cups	260	43
Early treatment	234	38.7
Good nutrition	143	23.7
Using separate rooms	201	33.3
Closing windows	107	17.7
Don’t know	35	5.8
Is TB treatable?		
Yes	329	54.5
No	167	27.6
Don’t know	108	17.9
Treatment method		
Herbal remedies	64	10.6
Home rest without treatment	99	16.4
Modern drugs	302	50.0
Self-treatment	117	19.4
Don’t know	116	19.2
Others	11	1.8
Overall TB knowledge		
Good	85	14.1
Moderate	128	21.2
Poor	391	64.7

Upon investigating the attitude and stigma related to TB, most respondents considered TB a somewhat serious disease (43.2%). In relation to their specific area, the majority thought TB to be a somewhat serious problem (34.4%). When asked how they would react if they were to be diagnosed with TB, fear and surprise were the most frequent choices (31.5% and 22.7%, respectively), and hopelessness was the least (7.1%). The overall attitude was poor in 89.9% of the participants. For more data on attitude, refer to Table 3.

**Table 3:** Participant attitudes and stigmatization of tuberculosis and patients with tuberculosis. TB: tuberculosis

Attitude	Number	Percentage
How serious is TB?		
Very serious	226	37.4
Somewhat serious	261	43.2
Not very serious	89	14.7
Don’t know	28	4.6
How serious a problem do you think TB is in your area?		
Very serious	117	19.4
Somewhat serious	208	34.4
Not very serious	201	33.3
Don’t know	78	12.9
Do you think you can get TB?		
Yes	142	23.5
No	224	37.1
Don’t know	238	39.4
What would be your reaction if you found out that you have TB?		
Fear	190	31.5
Surprise	137	22.7
Shame	80	13.2
Sadness	110	18.2
Hopelessness	43	7.1
Others (acceptance, taking proper medication)	44	7.3
Are some people more likely to become infected with TB than others?		
Yes	247	40.9
No	175	29.0
Don’t know	182	30.1
If yes, who is more likely to be infected?		
Men	58	23.5
Women	20	8.1
Both men and women	55)	22.3
Children	28	11.3
Very old people	86	34.8
Do you know people who have/had TB?		
Yes	222	36.8
No	319	52.8
Don’t know	63	10.4
What is your feeling towards people with TB disease?		
“I feel compassion and desire to help.”	139	23.0
“I feel compassion but stay away from them.”	179	29.6
“It is their problem and I cannot get TB.”	85	14.1
“I fear them because they may infect me.”	88	14.6
“I have no particular feeling.”	101	16.7
Others	12	2.0
In your community, how is a person who has TB usually regarded/treated?		
Most people reject them	86	14.2
Most people are friendly, but they generally try to avoid them	193	32.0
Mostly support and help them	230	38.1
Others (I don’t know, don’t give them specific attention)	95	15.7
Overall attitude		
Good	14	2.3
Moderate	47	7.8
Poor	543	89.9

Regarding participant practices pertaining to TB, most respondents said they would seek professional help by visiting a healthcare facility if they experienced symptoms of TB (61.4%), while others said they would go to pharmacies, traditional healers, or pursue self-treatment options (17.7%, 13.1%, and 7.8%, respectively). Most of those who chose options other than healthcare facilities explained that their behavior was due to not knowing where to go, not wanting to find something wrong, and their inability to leave work (20.9%, 20%, and 18%, respectively). Therefore, 59.4% of the respondents had poor knowledge of the proper practice to follow, 30.1% had moderate knowledge, and only 10.4% had good knowledge of the right practices for TB. More data on practice are provided in Table 4.

**Table 4:** Participants’ practices related to tuberculosis. TB: tuberculosis

Practice	Number	Percentage
Who would you talk to about your illness if you had TB?		
Doctor or another medical worker	283	46.9
Spouse	83	13.7
Parent	115	19.0
Close friend	94	15.6
No one	29	4.8
What would you do if you thought you had symptoms of TB?		
Go to a health facility	371	61.4
Go to a pharmacy	107	17.7
Go to traditional healers	79	13.1
Pursue self-treatment options (herbs, etc.)	47	7.8
If you had symptoms of TB, at what point would you seek medical help?		
When treatment on my own does not work	57	9.4
When TB symptoms last for two or more weeks	111	18.4
As soon as I realize TB symptoms	220	36.4
I would go to a health facility or contact a doctor	206	34.1
I don’t know	10	1.7
If you would not go to the health facility, what is the reason?		
I didn’t refuse to go to the hospital	151	25.0
Not sure where to go	126	20.9
Cost	108	17.9
Difficulties with transportation/distance	65	10.8
Do not trust medical workers	44	7.3
Do not like the attitude of medical workers	30	5.0
Cannot leave my work (overlapping work hours with medical facility working hours)	109	18.0

Participants’ gender played a major role in the results, as women were less likely to have poor knowledge in comparison to men (0.62, 95% CI, 0.44-0.87). Participants over 50 years old had substantially less knowledge about TB compared with participants 18-28 years old (7.61, 95% CI, 4.35-13.32). In addition, non-Saudi residents had less knowledge compared with Saudi residents (45.849, 95% CI, 18.475-113.78). Level of education also played a substantial role; university graduates had the most knowledge about TB compared with participants with no formal education and other education levels (0.052, 95% CI, 0.01-0.40). For more data on variables predicting poor TB knowledge, see Table 5.

**Table 5:** Variables predicting poor knowledge of tuberculosis. OR: odds ratio; AOR: adjusted odds ratio; R: reference category; CI: confidence interval *: P-value <0.05

Variable	OR (95% CI)	AOR (95% CI)
Sex		
Male	R	R
Female	0.62 (0.44-0.87)*	0.31 (0.20-0.53)*
Age (years)		
18–28	R	R
29–39	4.13 (2.35-7.22) *	2.09 (0.94-4.66)
40–50	6.06 (3.91-9.39) *	3.12 (1.43-6.82) *
Over 50	7.61 (4.35-13.32) *	3.99 (1.65-9.65) *
Nationality		
Saudi	R	R
Non-Saudi	45.849 (18.475-113.78)*	31.55 (11.43-87.09) *
Educational level		
No formal education	R	R
Elementary school	1.44 (0.09-24.63)	4.15 (0.21-81.21)
Intermediate school	1.53 (0.13-17.86)	2.65 (0.20-34.51)
High school	0.22 (0.028-1.70)	0.92 (0.11-8.16)
University graduate	0.052 (0.01-0.40) *	0.54 (0.06-4.58)
Monthly income (SAR)		
Less than 1,000	R	R
1,000–3,000	1.553 (0.89-2.71)	0.79 (0.37-1.66)
4,000–7,000	8.80 (4.49-17.24) *	1.08 (0.43-2.74)
8,000–10,000	9.97 (5.50-18.08) *	1.75 (0.72-4.23)
More than 10,000	1.19 (0.73-1.92)	0.33 (0.14-0.79) *
Presence of chronic diseases		
No	R	R
Yes	4.24 (2.66-6.76) *	2.90 (1.62=5.21) *

## Discussion

This study demonstrates that most participants (64.7%) had poor knowledge of TB, and only 21.2% had a moderate level of knowledge. In comparison, a cross-sectional study done in the eastern and western regions of Saudi Arabia reported poor knowledge of TB among 81.9% of participants. Another study in the country had a similar result at 74.9% [10,11]. Research conducted in Libya with a large sample also demonstrated limited knowledge, indicating the need for education on this subject [12]. However, most participants in our study had heard about TB (65.6%) and knew that bacteria cause it (48%), which is somewhat satisfactory in comparison to a study conducted in Riyadh revealing that only 35% of the participants knew the cause of TB [13]. The different study locations may explain this variation because TB is more prevalent in Makkah, affecting Saudis and non-Saudis equally. In a study conducted in Jeddah, 51% denied that TB is an infectious disease [14], contrasting with the results of the present study (29%), as well as studies conducted in Pakistan and Iraq [15,16]. This knowledge gap has an impact on the population of Makkah, mainly because it is an endemic area for TB. This highlights the necessity for educating the population, and, more importantly, educating pilgrims during the Hajj and Umrah (also known as the lesser pilgrimage) seasons to minimize the spread of the disease and correct misconceptions about it. There are many ways to provide education on TB. First, through social media which is a significant information source that is accessible to most of the population; and, second, through health education campaigns illustrating the seriousness of TB and the importance of seeking treatment while not promoting stigmatization. Regarding risk factors for poor knowledge about TB, our results revealed an association between poor TB knowledge and age and level of education. People over 50 tend to have less knowledge than those under 29 years old, and people with higher levels of education tend to have good knowledge of the subject. Many studies with similar results have reported an association between having a good level of knowledge and younger age and higher levels of education [17,18]. In our study, more participants who were non-Saudi and had low monthly income reported poor knowledge of TB. In a study conducted in Ethiopia, most respondents were delayed in seeking care because of cost and difficulties with transportation [19]. All of these factors play a significant role in the knowledge gap and misconceptions about the disease. Healthcare policy managers must strengthen health education efforts, especially targeting the younger generation and those with less education and low monthly income. Findings from the current study also revealed that women were less likely to have poor knowledge than men regarding TB, which might be because they had greater concern for their health or more contact with the media, a significant source of information for TB. Overall attitude was negative in most participants, corresponding to the results of studies conducted in Malaysia and Ethiopia [20,21]. However, in a study among women in Tabuk, Saudi Arabia, more than two-thirds of the studied population had a positive attitude toward TB despite gaps in knowledge and practice [22]. Furthermore, a significant number of participants in our study had stigmatizing thoughts toward people with TB and would avoid them because of their illness. Such a perspective might have considerable effects socially and mentally on the carriers of the disease. For example, a study done in Ghana revealed that most community members believe that TB patients should not be part of society [23]. In addition, a study conducted in Nepal found a link between TB and causes of discrimination such as poverty and low caste [24]. The study also reported that the participants believed TB was a divine punishment. These findings indicate the need to strengthen health education activities, including information, education, and communication.

On overall knowledge of practice, 59.4% of the participants showed poor results, while only 30.1% had moderate knowledge of good practice. Results showed that 20.9% were unsure where to go if they had TB symptoms, and another 20% feared knowing that they had the disease. Similar reasons were mentioned in southwest Ethiopia and Vietnam [21,25]. However, a study in Riyadh revealed that 67.6% of the general population had good practice knowledge, which could be a result of their high level of education and good healthcare­-seeking behavior [13].

Study limitations

This study has a few limitations. First, recall bias may have influenced results. Second, the study included only one region in Saudi Arabia and, hence, cannot be generalized to the whole country.

## Conclusions

Poor knowledge appears to be more prevalent among men than women and individuals over 50 years old. Moreover, more non-Saudi residents have poor knowledge, attitudes, and practices related to TB. More campaigns, whether in real life or through online platforms in association with the Ministry of Health, should be considered to raise awareness regarding TB.
